# Comprehensive circRNA Expression Profile and Construction of circRNAs-Related ceRNA Network in a Mouse Model of Autism

**DOI:** 10.3389/fgene.2020.623584

**Published:** 2021-02-16

**Authors:** Ji Wang, Zhongxiu Yang, Canming Chen, Yang Xu, Hongguang Wang, Bing Liu, Wei Zhang, Yanan Jiang

**Affiliations:** ^1^Yangzhou Maternal and Child Health Hospital, Yangzhou, China; ^2^Harbin Children's Hospital, Harbin, China; ^3^Xuzhou Children's Hospital, Xuzhou Medical University, Xuzhou, China; ^4^School of Civil Engineering, Northeast Forestry University, Harbin, China; ^5^Translational Medicine Research and Cooperation Center of Northern China, Heilongjiang Academy of Medical Sciences, Harbin, China; ^6^Department of Pharmacology (State-Province Key Laboratories of Biomedicine- Pharmaceutics of China, Key Laboratory of Cardiovascular Research, Ministry of Education), College of Pharmacy, Harbin Medical University, Harbin, China

**Keywords:** autism, circular RNA (circRNA), RNA sequencing (RNA-Seq), ceRNA network, *in silico* analysis

## Abstract

Autism is a common disease that seriously affects the quality of life. The role of circular RNAs (circRNAs) in autism remains largely unexplored. We aimed to detect the circRNA expression profile and construct a circRNA-based competing endogenous RNA (ceRNA) network in autism. Valproate acid was used to establish an *in vivo* model of autism in mice. A total of 1,059 differentially expressed circRNAs (477 upregulated and 582 downregulated) in autism group was identified by RNA sequencing. The expression of novel_circ_015779 and novel_circ_035247 were detected by real-time PCR. A ceRNA network based on altered circRNAs was established, with 9,715 nodes and 150,408 edges. Module analysis was conducted followed by GO and KEGG pathway enrichment analysis. The top three modules were all correlated with autism-related pathways involving “TGF-beta signaling pathway,” “Notch signaling pathway,” “MAPK signaling pathway,” “long term depression,” “thyroid hormone signaling pathway,” etc. The present study reveals a novel circRNA involved mechanisms in the pathogenesis of autism.

## Introduction

Autism spectrum disorder (ASD) is a multifactorial neurodevelopmental disorder diagnosed mainly during early life onset, which is often combined with attention deficit/hyperactivity disorder, mental disorder, and intellectual disability (Vahabzadeh et al., 2018; Valiente-Palleja et al., 2018; Miryounesi et al., 2019). According to the Diagnostic and Statistical Manual of Mental Disorders, 5th ed (DSM-5), it is characterized by impaired social interactions and elevated stereotyped activities, and the global prevalence is about 1% (Lai et al., 2014). Autism prevalence is increasing globally. According to the latest data of Autism and Developmental Disabilities Monitoring (ADDM) network in 2018, ASD prevalence was 1 in 54 (https://www.cdc.gov/ncbddd/autism/data.html). Early screening and diagnosis are very important to improve the outcome of autism patients. Unfortunately, primary health care professionals are usually unaware of the early manifestations of autism, and the gold standard diagnostic tools, autism Diagnostic Interview Revised (ADI-R) and Autism Diagnostic Observation Schedule (ADOS), are time-consuming and expensive. The pathogenesis of autism is associated with genetic and epigenetic alterations (Olde Loohuis et al., 2015; Turner et al., 2017). However, there is no specific autism therapeutic drug because the pathogenesis mechanism of autism is still not fully clarified.

Circular RNAs (circRNAs) are a kind of non-coding RNAs with circular structure, which are rapidly becoming considered as critical regulators of gene expression networks (Salzman et al., 2013). Furthermore, studies have proved that circRNAs are widely expressed in tissue-and-developmental-stage-specific patterns, and a fraction of them displays conservation across species (Rybak-Wolf et al., 2015). Importantly, a general phenomenon was discovered that circRNAs bind to miRNAs, acting as miRNA sponges, and thereby affect the expression of target genes (Hansen et al., 2013). Several studies have shown that circRNAs are more abundantly expressed in the brain than other tissues in mammals (You et al., 2015; Li et al., 2017). CircRNAs also show dynamic expression during neurogenesis, synaptogenesis, and neuronal differentiation (Izuogu et al., 2018). These findings indicated that circRNAs are likely to play functional roles in neuron development and diseases.

Even though studies revealed that circRNAs are highly abundant in the brain, the expression profile, function, and mechanism of most circRNAs in autism are still largely unelucidated. Chen et al. (2020) found a series of autism-associated circRNAs in autism cortex samples. Our study aimed to determine the circRNA profile in brain tissues from valproic acid-induced mouse autism model and analyze their function and potential mechanism.

## Materials and Methods

### Establishment of A Mouse Model of Autism

C57BL/6 mice were bought from Liaoning Changsheng Biotechnology Co., Ltd. (Liaoning, China). Exposure to some neurotoxic drugs may induce fetal nervous system development disorders. Valproate acid (VPA) is a widely used drug to induce autism (Baronio et al., 2018; Eissa et al., 2018). The animal model of autism was established as previously described (Zheng et al., 2019). Briefly, female adult mice were mated to males overnight. Pregnant mice were injected with valproic acid (Sigma, Ronkonkoma, NY, USA) 500 mg/kg at embryonic day 12.5 (E12.5). Mice in control group received a considerable amount of normal saline. All pregnant mice were allowed to give birth naturally, and the 1st day of birth was recorded as postnatal day 1 (P1). Pups were weaned at the 23rd day (p23) after birth. The animal protocol was approved by the Harbin Children's Hospital Animal Care and Use Committee (JJ2017ZR0484).

### Repetitive Self-Grooming Behavior Measurement

On the 32nd day after birth, mice were placed in open field box for 10 min to adapt to the environment, then start timing for 10 min to observe mice behavior (Onaolapo et al., 2017). The number of body cleaning with paws and face-washing actions was calculated.

### Social Interaction Test

Social interaction test was used to assess active interaction time in a test mouse with a novel mouse. (1) Adaptation stage: Gently put the tested mice into the device (material: acrylic glass, 40 × 40 × 30 cm, covered with 2–3 cm thick bedding), and let them move freely inside 10 min. (2) Test stage: Take out the test mice that have just adapted, and put them in the test mice and interactive mice (the same strains of mice that have the same sex and age as the test mice that have not been raised in the same cage). The number of social behaviors (physical contact and following peers) and non-social behaviors (investigation) of the mice were recorded within 10 min.

### Real-Time PCR Analysis

TRIzol reagent (Invitrogen, CA, USA) was used to extract total RNA. A reverse transcriptase kit (Roche, Mannheim, Germany) was used to synthesize first-strand cDNA. Reverse transcription was performed using HiScript® II Q RT SuperMixfor qPCR (Vazyme Biotech Co., Ltd., Nanjing, China). Real-time PCR analysis was performed on an ABI step one plus system (Applied Biosystems, CA, USA). Primer sequences were as follows: novel_circ_000430, 5′-ACCCGTCTTCAGTCTCCGT-3′ (forward), 5′-AATATCACCCACACCCTCAGC-3′ (reverse); novel_circ_015779, 5′-CTCTGCCTGGTGCTGGTATTG-3′ (forward), 5′-ATGTAACTCTCTCCCTCCCCTG-3′ (reverse); novel_circ_063340, 5′-GCTTACCGTGGAGATGTTTGAC-3′ (forward), 5′-CGCCTTCTCCAACACCTCA-3′ (reverse); β-actin, 5′-ACCCATTCTCTGTCTCGCAC-3′ (forward), 5′-ATCGTCACCCCCAAAACCTG-3′ (reverse). β-actin was used as an internal control. Target gene expression was analyzed using the 2^−ΔΔCT^ method.

### RNA Sequencing

RNA sequencing was conducted by Gene Denovo Biotechnology Co. (Guangzhou, China). The circRNA were identified using find_circ (Memczak et al., 2013). The edge R package (http://www.rproject.org/) was used to identify differentially expressed circRNAs. CircRNAs with *P* < 0.05 and |log2FoldChange| > 1 in a comparison between control and autism groups was identified as differentially expressed.

### CircRNA-miRNA-mRNA Network Construction

The miRNA binding sites of annotated circRNAs in circBase was predicted by StarBase (v2.0) (Li et al., 2014). The miRNA binding sites of novel circRNAs were predicted by Mireap, Miranda (v3.3a) (Betel et al., 2010), and TargetScan (v7.0) (Agarwal et al., 2015). Subsequently, miRTarBase (v6.1) (Hsu et al., 2011) was used to predict mRNAs that interact with circRNAs through miRNAs. The established competing endogenous RNA (ceRNA) network was visualized by Cytoscape (Version 3.7.2) (Kohl et al., 2011).

### Gene Ontology and Pathway Enrichment Analysis

Significantly enriched Gene Ontology (GO) terms in source genes comparing to the genome background were defined by hypergeometric test (Ashburner et al., 2000). Kyoto Encyclopedia of Genes and Genomes (KEGG) database was used to perform enrichment analysis (Kanehisa et al., 2008).

### Statistical Analysis

Data are expressed as mean ± SEM and compared using Student's *t*-test. A two-tailed *P* < 0.05 was required for significance.

## Results

### A Mouse Model of Autism Was Successfully Established

**Figure 1:**
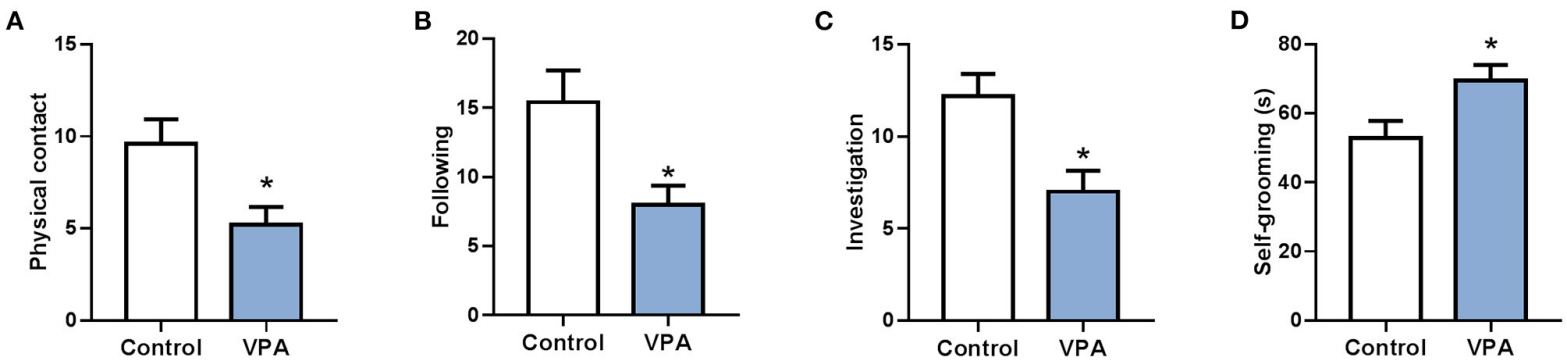
A mouse model of autism was successfully established. **(A)** The social interaction test (physical contact). **(B)** The social interaction test (following). **(C)** The social interaction test (investigation). **(D)** Repetitive self-grooming behavior. *P < 0.05 vs. Control; *n* = 4–5.

To analyze different expression of circRNA in the brain of autistic mice, we established a mouse model of autism and verified the model through behavioral testing. The animal model of autism was induced as previously described (Zheng et al., 2019). On the 28th day after birth, their behavioral ontogeny was evaluated using the social interaction test, repetitive self-grooming behavior. Results showed that the offspring mice in the model group exhibited autism-like behavioral abnormalities (Figure 1).

### Expression Profile of circRNAs in A Mouse Model of Autism

**Figure 2:**
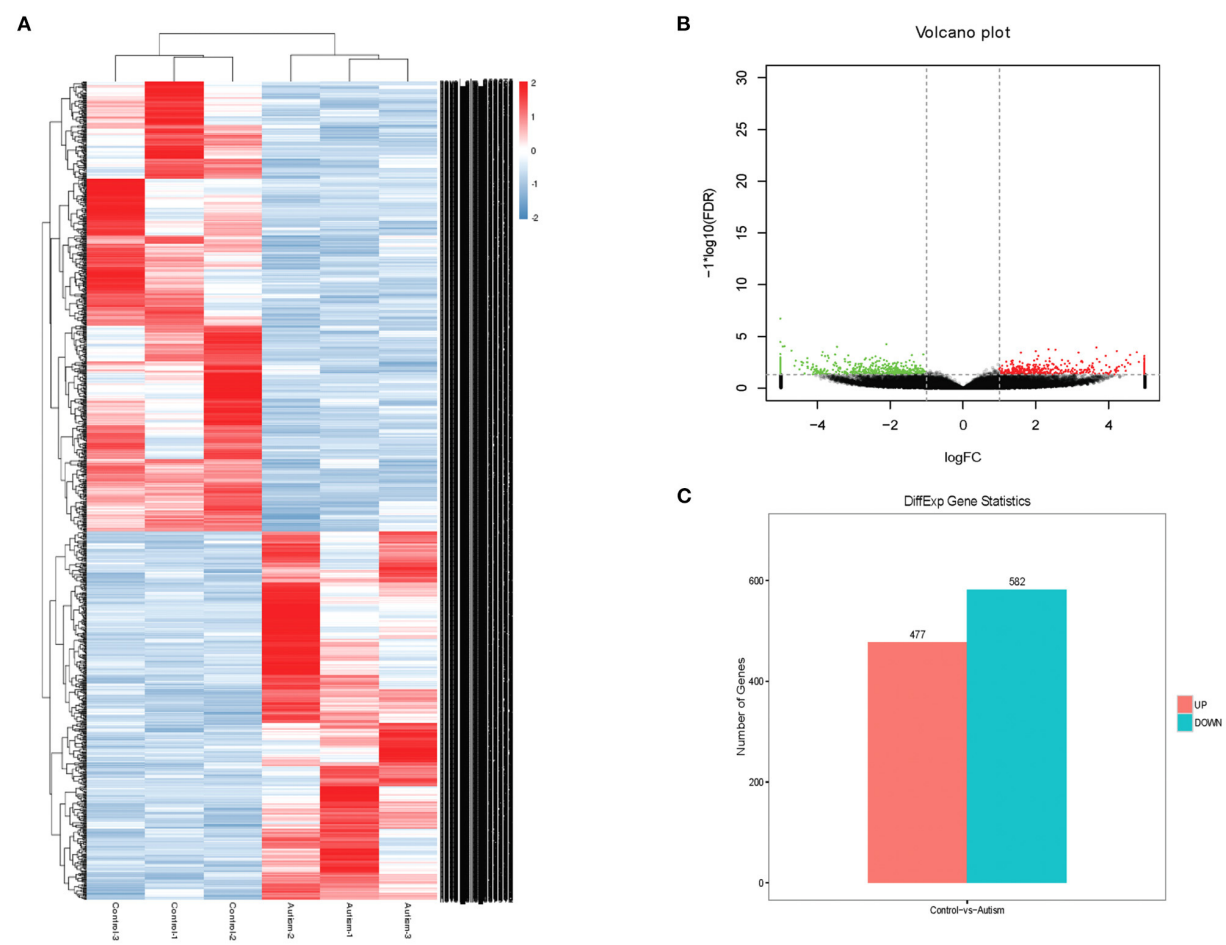
Differentially expressed circRNAs in brain from autism mice. **(A)** Heat map. **(B)** Volcano plots. **(C)** The number of differentially expressed circRNAs in a mouse model of autism.

The expression profile of circRNAs in brain tissues from autism mouse model and corresponding controls was evaluated using RNA sequencing. The hierarchical cluster analysis of circRNA is shown in Figure 2A. The volcano plots of circRNAs are shown in Figure 2B. A total of 1,059 altered circRNAs were identified in the autism group, with 477 upregulated and 582 downregulated (GSE163904, Figure 2C, Table 1, and Supplementary Material).

**Table 1:** Biological information regarding the top 10 upregulated and downregulated circRNAs in autism mouse model.

	**circRNA**	**log2(Fold change)**	***p*-value**
Upregulated	novel_circ_015779	14.07865	1.88E-03
	novel_circ_063340	13.83892	1.82E-03
	novel_circ_000430	13.75961	1.23E-03
	novel_circ_052619	13.754	3.88E-03
	novel_circ_011355	13.72869	6.55E-03
	novel_circ_002576	13.67244	7.79E-04
	novel_circ_013547	13.65162	9.93E-03
	novel_circ_058403	13.6008	1.33E-03
	novel_circ_028696	13.58711	8.98E-03
	novel_circ_018711	13.57057	1.20E-02
Downregulated	novel_circ_014536	−14.9586	1.90E-07
	novel_circ_066322	−14.168	3.44E-05
	novel_circ_022010	−13.7173	5.14E-04
	novel_circ_056205	−13.5461	1.36E-03
	novel_circ_001586	−13.5253	8.62E-03
	novel_circ_065910	−13.5045	1.93E-03
	novel_circ_000370	−13.4837	1.10E-03
	novel_circ_018104	−13.479	1.09E-03
	novel_circ_012200	−13.436	2.96E-03
	novel_circ_061645	−13.392	1.61E-02

### CircRNA Expression Verified by Real-Time PCR

**Figure 3:**
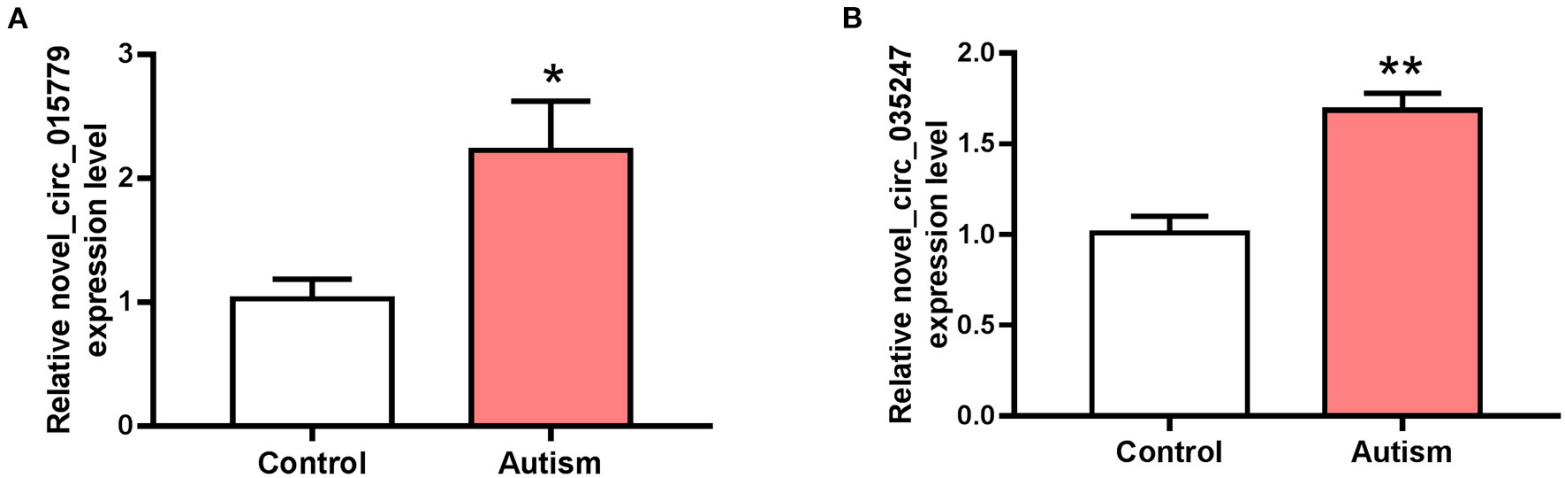
The expression of novel_circ_015779 and novel_circ_035247 in control and autism groups. **(A)** The expression of novel_circ_015779. **(B)** The expression of novel_circ_035247. *P < 0.05, **P < 0.01 vs. Control, *n* = 3.

The expressions of novel_circ_015779 and novel_circ_035247 were detected by real-time PCR assay. The expression levels of novel_circ_015779 and novel_circ_035247 were upregulated in the autism group (Figure 3). These results were in accordance with that in RNA sequencing.

### The ceRNA Network Construction

**Figure 4:**
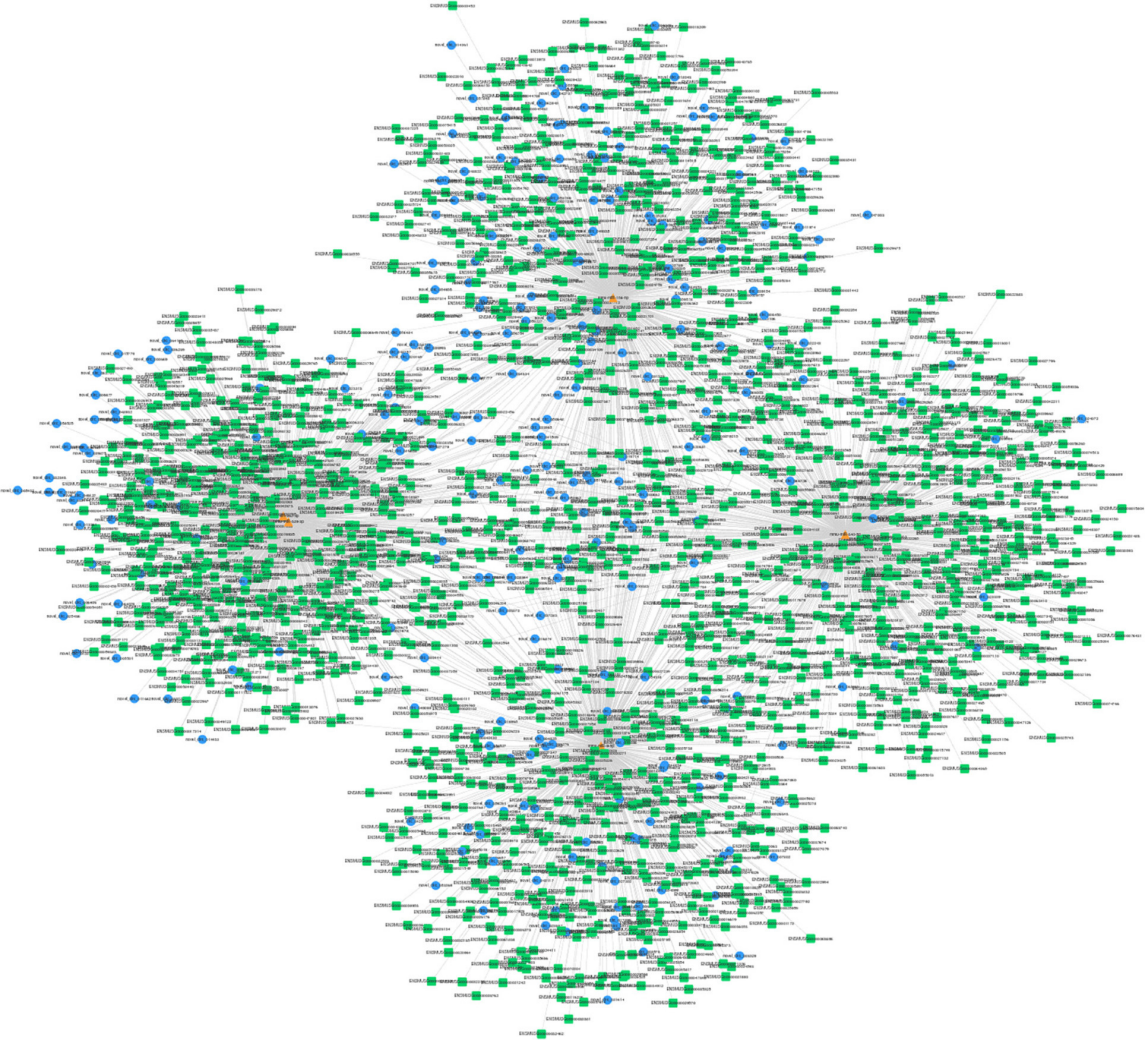
Top 5 miRNA-related circRNAs-miRNA-mRNA ceRNA network. Circular blue, triangular orange, and square green nodes represent circRNAs, miRNAs, and mRNAs, respectively.

**Figure 5:**
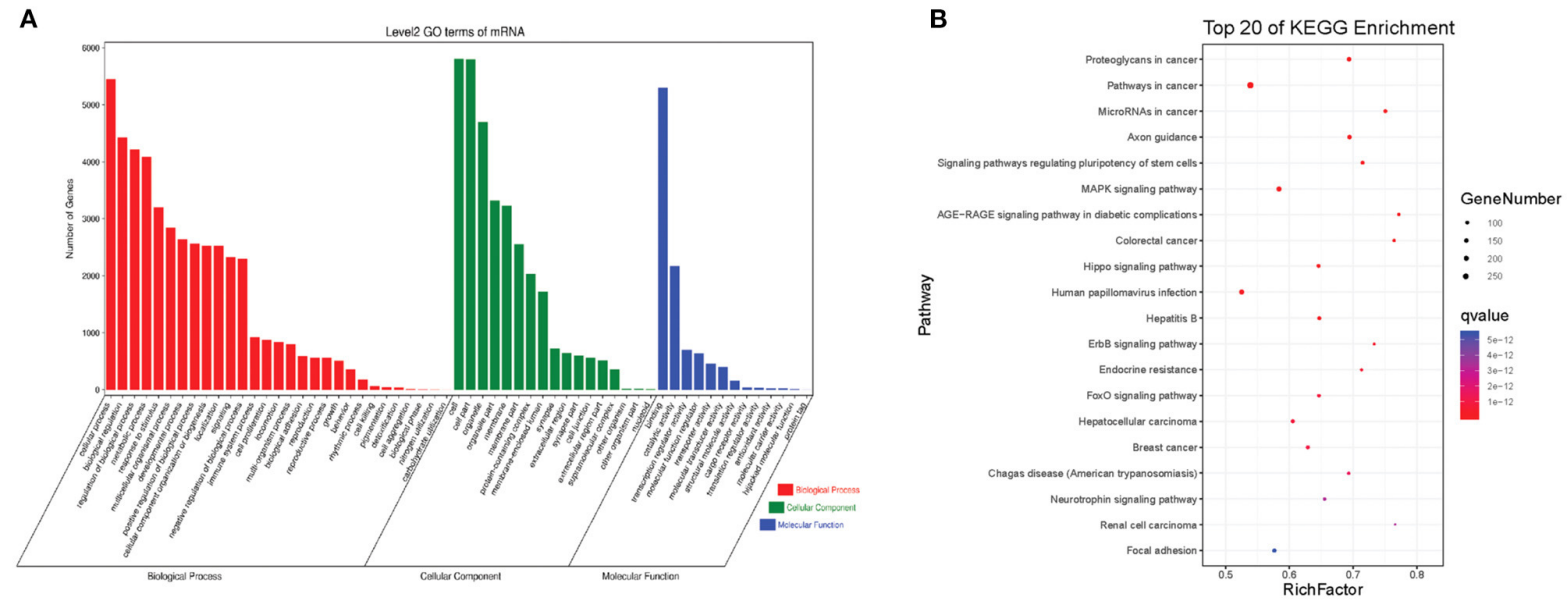
The GO and KEGG pathway enrichment of autism-related circRNAs-miRNA-mRNA ceRNA network. **(A)** GO enrichment. **(B)** KEGG pathway enrichment. The color bar represents the *q*-value.

The interactions between circRNAs/mRNA and miRNAs were predicted using StarBase (v2.0), Miranda (v3.3a), TargetScan (v7.0), and miRTarBase (v6.1). Then, a ceRNA network was constructed using differentially expressed circRNAs and bioinformatic predication results. The established circRNAs-miRNA-mRNA ceRNA network contains 9,715 nodes (including 1,059 circRNAs, 6,730 mRNA, and 1,926 miRNA) and 150,408 edges. A top 5 miRNA-based circRNA-miRNA-mRNA network is shown in Figure 4. The top 10 hub nodes are shown in Table 2. GO analysis showed that the ceRNA network was mainly associated with several biological processes, including “cellular process,” “biological regulation,” “regulation of biological process,” and “metabiological process.” Meanwhile, KEGG pathway analysis showed the ceRNA network was mainly associated with “axon guidance,” “MAPK signaling,” “Hippo signaling pathway,” and “ErbB signaling pathway” (Figure 5).

**Table 2:** Hub nodes with top degrees in the ceRNA network.

**Rank**	**circRNA**	**miRNA**	**mRNA**
1	novel_circ_050499	mmu-miR-15a-5p	Tbc1d24
2	novel_circ_021126	mmu-miR-340-5p	Gabpb2
3	novel_circ_002396	mmu-miR-362-3p	Ybey
4	novel_circ_054171	mmu-miR-9-5p	Chic1
5	novel_circ_002378	mmu-miR-329-3p	Hecw1
6	novel_circ_025446	mmu-miR-7b-5p	Cd28
7	novel_circ_028364	mmu-miR-181a-5p	Asxl2
8	novel_circ_036195	mmu-miR-466l-5p	Ppm1k
9	novel_circ_041509	mmu-miR-466i-5p	Kcnk6
10	novel_circ_000669	mmu-miR-466k	Car5b

### Module Analysis of the ceRNA Network

**Figure 6:**
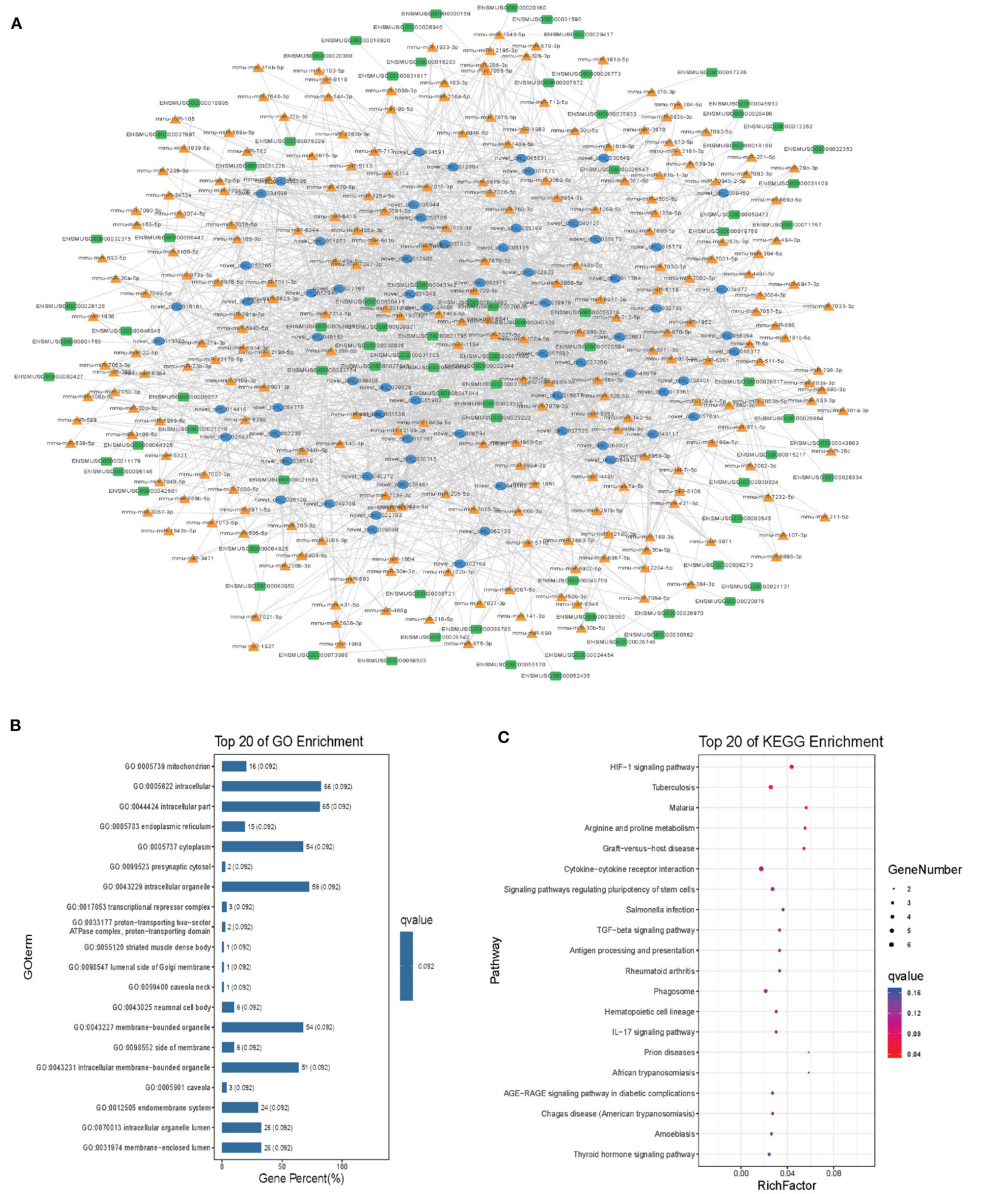
Module 1 from the autism-related ceRNA network. **(A)** Module 1 network. **(B)** GO enrichment. **(C)** KEGG pathway enrichment. Circular blue, triangular orange, and square green nodes represent circRNAs, miRNAs, and mRNAs, respectively.

**Figure 7:**
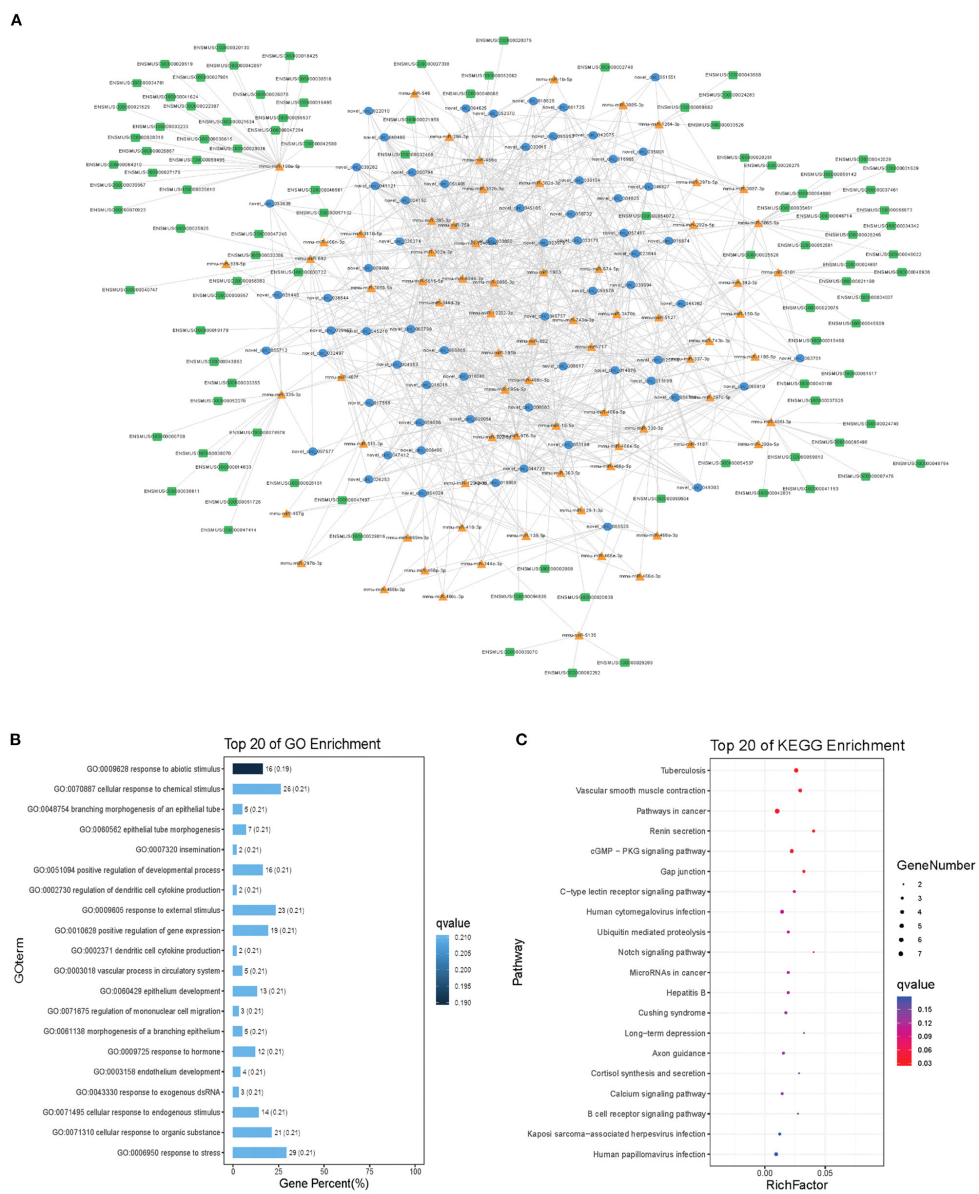
Module 2 from the autism-related ceRNA network. **(A)** Module 2 network. **(B)** GO enrichment. **(C)** KEGG pathway enrichment. Circular blue, triangular orange, and square green nodes represent circRNAs, miRNAs, and mRNAs, respectively.

**Figure 8:**
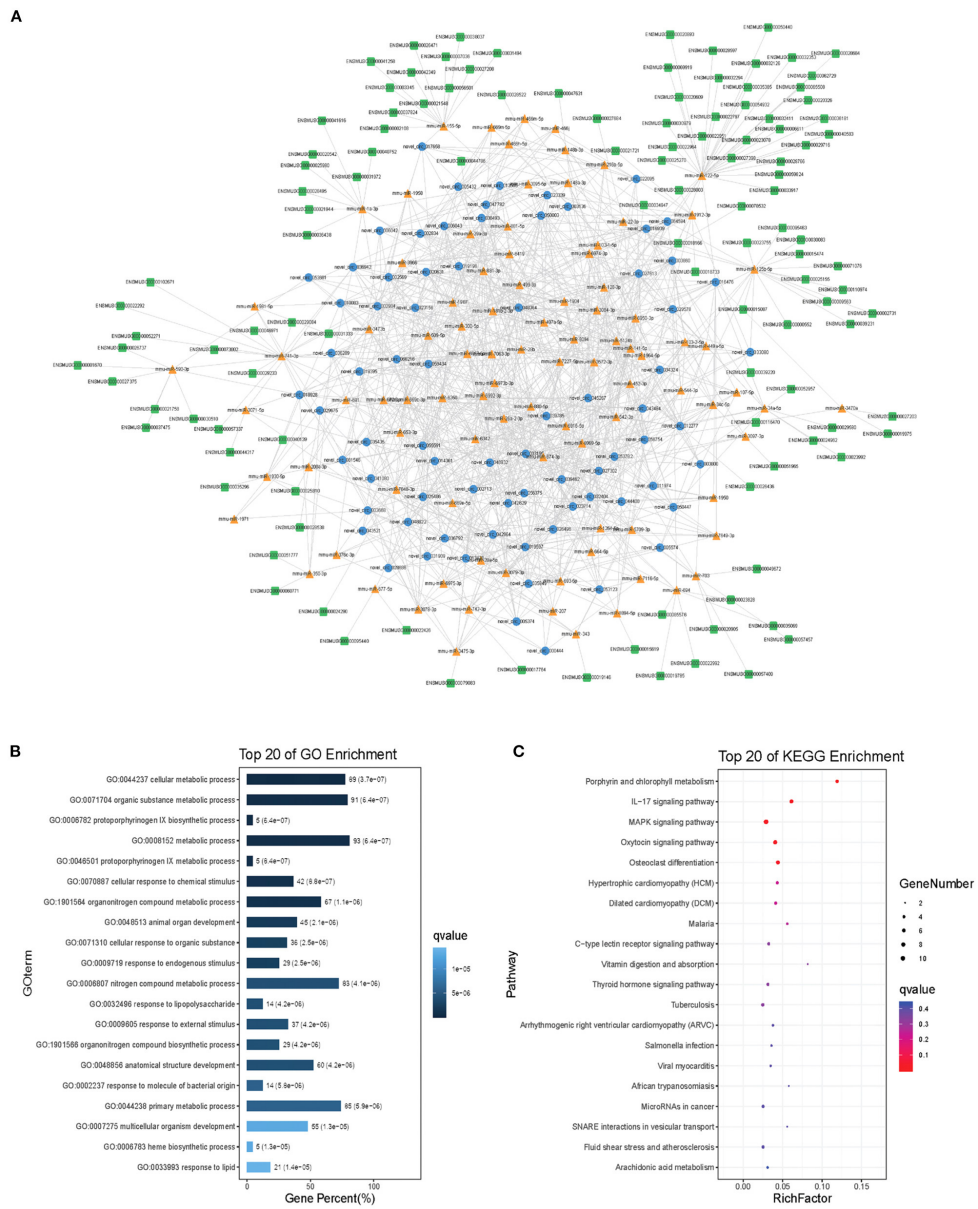
Module 3 from the autism-related ceRNA network. **(A)** Module 3 network. **(B)** GO enrichment. **(C)** KEGG pathway enrichment. Circular blue, triangular orange, and square green nodes represent circRNAs, miRNAs, and mRNAs, respectively.

Modules were screened using plug-in MCODE. GO and KEGG pathway enrichments were performed in the top three modules, respectively. Module 1 consists of 393 nodes (68 circRNAs, 239 miRNAs, and 86 mRNAs) and 1,508 edges, which are mainly associated with “HIF-1 signaling pathway,” “arginine and proline metabolism,” “TGF-beta signaling pathway,” “IL-17 signaling pathway,” etc. (Figure 6). Module 2 consists of 242 nodes (67circRNAs, 73 miRNAs, and 102 mRNAs) and 891 edges, which are mainly related to “renin secretion,” “cGMP-PKG signaling pathway,” “Notch signaling pathway,” “long term depression,” etc. (Figure 7). Module 3 consists of 289 nodes (75 circRNAs, 95 miRNAs, and 119 mRNAs) and 1,011 edges, which are mainly associated with “IL-17 signaling pathway,” “MAPK signaling pathway,” “C-type lectin receptor signaling pathway,” “thyroid hormone signaling pathway,” etc. (Figure 8).

## Discussion

The incidence of autism is still increasing, which seriously affects the quality of human life. Despite the research progress that has been made in recent years, the pathogenesis mechanisms of autism are still not fully clarified. The development of bioinformatics facilitated the investigation of disease mechanisms and therapeutic strategies (Peng et al., 2017, 2018; Zhou et al., 2019). Our study comprehensively elucidated circRNA expression profile in a mouse model of autism and constructed a circRNA-associated ceRNA network.

CircRNAs are a special kind of non-coding RNAs with circular structure. CircRNAs could exert biological function through a ceRNA mechanism (circRNA-miRNA-mRNA), circRNA–protein interaction, or regulate translation (Du et al., 2017; Sun et al., 2019; Yi et al., 2019). This kind of non-coding RNAs is involved in various diseases, including cancers, cardiovascular diseases, and neuronal diseases (Chen et al., 2017; Sekar et al., 2018; Yang et al., 2019). CircRNAs are shown to be associated with schizophrenia and cognitive dysfunction (Zhang et al., 2017; Mahmoudi et al., 2019). However, the expression and role of circRNAs in autism is still poorly understood. Therefore, we detected the circRNA expression profile using RNA sequencing in a mouse model of autism.

RNA sequencing identified a total of 1,059 differentially expressed circRNAs in the autism group. The expression level of novel_circ_015779 and novel_circ_035247 was detected by real-time PCR assay. The RNA sequencing and real-time PCR assay achieved similar results.

CircRNAs could act as a ceRNA and thus exert biological functions. Using bioinformatic methods, we predicted the interaction between circRNAs and miRNA and constructed an autism-related ceRNA network. The ceRNA network contains 150,408 edges and 9,715 nodes, including 1,059 circRNAs, 1,926 miRNA, and 6,730 mRNA.

We then performed GO and KEGG analysis. The autism-related ceRNA network was signaling pathways including “axon guidance,” “MAPK signaling,” “Hippo signaling pathway,” and “ErbB signaling pathway.” Among these signaling pathways, the role of “axon guidance” and “MAPK signaling” in autism has been proved (Suda et al., 2011; Rosina et al., 2019). It has been reported that aberrant axon-guidance protein expression was observed in the brains of people with autism, including the decreased expression of PLXNA4 and ROBO2 (Suda et al., 2011). And the disruption of the MAPK pathways correlates with severity in idiopathic autism (Rosina et al., 2019).

The hub nodes, take miR-142-3p, miR-142a-5p, and miR-142b for example, may play an important role in autism. Mor et al. (2015) found that miR-142a-5p and miR-142a-3p were upregulated in brain tissues of autism patients compared with that in controls. It has been reported that the upregulation of these two miRNAs could induce apoptosis (Lu et al., 2015; Kim et al., 2018). Apoptosis was also related to autism (Dong et al., 2018). Therefore, miR-142 cluster and its related circRNAs may be involved in the pathogenesis of autism, which needs further investigation. miR-9-5p was also a hub nod, which targets Dlg4 (encode PSD-95). The deletion or variation of Dlg4 was associated with autism (Feyder et al., 2010). In the ceRNA network, novel_circ_000370, novel_circ_050499, novel_circ_021126, etc. may regulate Dlg4 through miR-9-5p.

Subsequently, network modules analysis was conducted. The top three modules were all correlated with autism-related pathways including “TGF-beta signaling pathway,” “Notch signaling pathway,” “MAPK signaling pathway,” “long-term depression,” etc. Take “TGF-beta signaling pathway” as an example; TGF-β1 was upregulated in the brain of autistic patients (Vargas et al., 2005). Early and adult hippocampal TGF-β1 overexpression led to a series of autism-related behaviors (Depino et al., 2011). The circRNAs involved in these signaling pathways may play a regulatory role in autism.

Circulating miRNAs were aberrantly expressed in autism individuals compared with that in controls (Mundalil Vasu et al., 2014; Hicks et al., 2016; Jyonouchi et al., 2019). In the serum of individuals with autism, 13 miRNAs were differentially expressed, some of which showed diagnostic potential for patients with autism (Mundalil Vasu et al., 2014). Subsequent research achieved similar results, that serum miRNAs may be promising biomarkers for autism (Kichukova et al., 2017; Jyonouchi et al., 2019). Besides, 14 miRNAs were identified differentially expressed in salivary samples from autism patients, and most of them significantly correlate with Vineland neurodevelopmental scores, including miR-27a, miR-23a, and miR-628-5p (Hicks et al., 2016). However, the function of these miRNAs in autism remains largely uninvestigated. In our ceRNA network, we identified a series of potential signaling pathways involved in the pathogenesis of autism, which may be helpful to clarify the role of miRNAs in autism. Besides, circRNAs also exist in the circulation system, which may also serve as biomarkers for autism. The expression of circRNAs in serum from autism patients and animal models still needs further investigation.

In conclusion, our study provides the expression profile of circRNAs in a mouse model of autism, which promotes our understanding in the pathogenesis mechanism of autism.

## Data Availability Statement

The RNA sequencing results for this study could be found in the GEO repository with the accession number of GSE163904.

## Ethics Statement

The animal study was reviewed and approved by Harbin children's hospital Animal Care and Use Committee [JJ2017ZR0484].

## Author Contributions

JW, YJ, and WZ designed the study. HW and BL performed the animal experiment. ZY, CC, and YX analyzed the data. JW and YJ wrote the manuscript. All authors reviewed the manuscript.

## Conflict of Interest

The authors declare that the research was conducted in the absence of any commercial or financial relationships that could be construed as a potential conflict of interest.
